# Lemon juice pretreatment as a strategy to preserve the quality and enhance the texture of cooked potato slices of different sizes

**DOI:** 10.1016/j.fochx.2024.101800

**Published:** 2024-09-02

**Authors:** Alsadig Yahya, Abdeen Elkhedir, Mamoun A. Homaida, Yassin Haran, Ikhlas Galal-Eldin, Yassin Taha, Ezzalden Saleh

**Affiliations:** a Department of Food Science and Technology, Faculty of Agriculture and Natural Resources, University of Bakht Al-Ruda, Ed Dueim, Sudan.; b College of Food Science and Technology, Sudan University of Science & Technology, Khartoum 11115, Sudan.; c Sudanese Standards and Metrology Organization, Khartoum 11115, Sudan.

**Keywords:** Potato slices, Lemon juice pretreatment, Texture quality, Nutritional profile, SEM, FTIR, Volatile compounds, Citric acid (PubChem CID: 311), Ascorbic acid (PubChem CID: 54670067), Chlorogenic acid (PubChem CID: 1794427), Pectin (PubChem CID: 441476), Amylose (PubChem CID: 439174), Calcium ions (PubChem CID: 5460341), Carveol (PubChem CID: 439570), Decanal (PubChem CID: 8172), Methyl allyl disulfide (PubChem CID: 5366425), Furan 2-pentyl (PubChem CID: 5366233)

## Abstract

Potatoes are an important food crop worldwide and are rich in essential nutrients. However, cooking can reduce their nutritional value and alter their texture. This study aimed to investigate the impact of pretreating potato slices with lemon juice. The slices were immersed in 5% lemon juice solution for 3 h, rinsed with distilled water for another 3 h, then cooked at 100°C for 20 min. Findings revealed that lemon juice pretreatment (LJP) notably improved the texture, mouthfeel, and overall acceptability of the cooked potato slices of different sizes (CPS-Ds). Additionally, LJP significantly increased vitamin C and total phenolic contents, slightly decreased pH levels, and preserved the desired color of CPS-Ds. Consumer sensory evaluations also indicated a positive response to LJP samples, suggesting its potential application in the food industry. The study confirmed that LJP is an effective, sustainable, consumer-friendly, and cost-efficient technique for improving the quality of cooked potato slices.

## Highlights

• Lemon juice improves the texture, flavor, and nutrition of potato slices.
• Immersing potato slices in 5 % lemon juice significantly improves their quality.
• Lemon juice pretreatment acts as a natural food preservative for potatoes.
• Pretreatment increases vitamin C and phenolic contents in cooked potatoes.

## Graphical abstract

**Figure:**
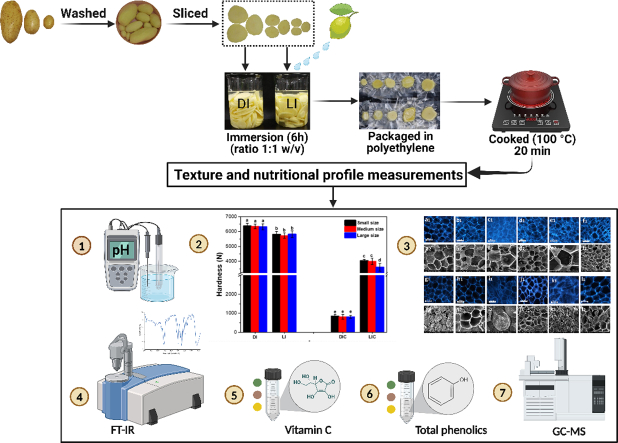

## 1 Introduction

Potatoes (*Solanum tuberosum* L.) are cultivated in over 100 countries worldwide and are considered one of the staple foods in human diets alongside maize, wheat, and rice (García-Segovia et al., 2008; Zhao et al., 2017). They also serve as an important raw material for various industries. Potatoes are among the most efficient sources of energy and contain essential nutrients such as vitamins, antioxidants, and minerals that support overall health. The average composition of a potato tuber includes starch (10–18 %) with an amylose content of 22–30 %, total sugars (1–7 %), protein (1–2 %), fiber (0.5 %), lipids (0.1–0.5 %), ascorbic acid (30 mg per 100 g, fresh weight (FW)), minerals (trace amounts), glycoalkaloids (1–3 mg per 100 g FW), carotenoids (50–100 μg per 100 g FW), and phenolic compounds (20–60 μg per 100 g FW). However, the thermal processing of potatoes greatly affects their texture (Zhao et al., 2017) and nutritional profile (Moens et al., 2021).

The texture of potatoes undergoes significant changes during thermal treatments, leading to the rupture of membranes and cell walls, loss of turgor pressure, and progressive starch gelatinization (Fuentes et al., 2014). Therefore, understanding how to avoid these problems during the cooking process is crucial for food scientists (Bordoloi et al., 2012). Recently, the potential application of food-grade organic acids to reinforce the hardness of potato slices during cooking has been reported (Liu, Bi, et al., 2020; Zhao et al., 2017). It has also been reported that pre-treating potato slices with lactic acid before boiling can prevent the degradation of the potato cell wall structure (Liu, Liu, et al., 2020). The mechanism for enhancing the texture of cooked potato slices (CPS) through acid-based pretreatment can be attributed to two factors. Firstly, the infiltration of acid into the potato cells inhibits the activity of poly-galacturonase and slows down the degradation of the potato cell wall structure. Secondly, the penetration of acid into the potato cells promotes the in situ gelation of pectin, which tightly binds the potato cells (Liu, Wen, et al., 2020). Additionally, acetic acid pretreatment has also been found to enhance the hardness of CPS (Zhao et al., 2017). Furthermore, calcium ions have been found to play a critical role in enhancing the texture of CPS by forming cross-links among pectin molecules (Andersson et al., 1994; Moens et al., 2021).

The nutritional quality of potatoes, such as vitamin C and total phenolics decreases significantly during thermal treatments (Park et al., 2020). Potatoes are an excellent source of ascorbic acid (Gong et al., 2022; Gutiérrez-Quequezana et al., 2018). However, cooking them leads to a significant loss of vitamin C, regardless of the cooking method used. This is because vitamin C is sensitive to heat treatments, easily dissolves in water, and is not stable at high temperatures (Tian, Chen, Lv, et al., 2016). The preparation method is also another factor that can greatly affect the ascorbic acid content of potatoes (Fabbri & Crosby, 2016). While most publications have reported losses of vitamin C during various cooking methods, the degree of loss varies significantly, even when the same cooking methods are used. Some losses range from 10 % to 20 %, while others are as high as 70 % to 80 % (Tian, Chen, Ye, & Chen, 2016).

Potatoes are a potential source of polyphenols, which have health benefits. Phenolic acids like chlorogenic acid, ferulic acid, neochlorogenic acid, caffeic acid, syringic acid, gallic acid, protocatechuic acid, coumaric acid, and vanillic acid play important roles in preventing diseases. These compounds contribute to the antioxidant, anti-carcinogenic, anti-inflammatory, anti-obesity, and anti-diabetic activities of potatoes. The pharmaceutical and nutraceutical industries benefit from these biological activities (Anitha & Manivannan, 2023). However, these essential compounds degrade when exposed to heat, reducing the nutritional value of potatoes. Tian, Chen, Lv, et al. (2016) reported that cooking significantly reduces total phenolics in potatoes compared to uncooked ones, which contain 209 ± 35.7 mg gallic acid equivalents (GAE) per 100 g FW. The decrease in total phenolics may be attributed to water-soluble phenols leaching into the cooking water and the breakdown of phenolic substances during heat processing (Fang et al., 2022). Additionally, phenolic compounds participate in the Maillard reactions during processing, leading to an increase in Maillard reactions products and therefore decrease in the phenolic levels in CPS (Tian, Chen, Ye, & Chen, 2016).

Lemon (*Citrus limon* L.) is an important crop in fruit production and a common ingredient in cooking due to its tart flavor. Its high acidity extends the shelf life of foods by inhibiting bacterial growth. Lemon juice can be used in sweet and savory dishes, as well as in marinades and as a meat tenderizer. It is also used as a natural preservative in canned goods, jams, jellies, and pickles by increasing acidity and preventing microbial growth. Lemon juice can also be used as a substitute for vinegar in recipes such as salsa, providing a milder acidic flavor (Ayonga, Ondieki, & Ronoh, 2023; Sarkar et al., 2024; Vujčić Bok, Šola, & Rusak, 2022). To date, no studies have yet been reported on using LJP to enhance the texture and nutritional profile of CPS-Ds. We hypothesized that LJP might synergistically reduce heat softening by modifying plant cell walls and dissolving pectin polymers in cell adhesion. Therefore, this study aimed to investigate the effectiveness of 5 % LJP in maintaining cell wall integrity and enhancing the nutritional value of CPS-Ds. The pH value, sensory attributes, color value, hardness, microstructures analysis, vitamin C content, total phenolic content, volatile compounds, and absorption spectra of CPS-Ds were thereafter measured. We do believe that our findings could contribute to the development of high-quality vegetables with extended shelf life, making them suitable for cost-effective industrialization while remaining consumer-friendly.

## 2 Materials and methods

### 2.1 Raw materials

Potato tubers used in this study included small-size (length ∼ 62 mm, width ∼ 51 mm, weight ∼ 148 g), medium-size (length ∼ 93 mm, width ∼ 69 mm, weight ∼ 378 g), and large-size (length ∼ 124 mm, width ∼ 91 mm, weight ∼ 718 g). 2 Kg from each tuber size were purchased from a vegetable market (Wuhan, China) and utilized for the experiments without further storage.

### 2.2 Chemicals

Chemicals and reagents used in the study included isopropyl acetate, ethanol, agarose, formaldehyde, paraformaldehyde, oxalic acid, sodium bicarbonate, methanol, gallic acid, cyclohexane, and other solvents with analytical grades were acquired from Aladdin Co., (Shanghai, China). MillQ-H_2_O was used in this study.

### 2.3 Sample preparation

Potato tubers of different sizes (small, medium, and large) were washed, peeled, and sliced with a potato slicer (Crypto Peerless Ltd.). The samples were then divided into two groups. One group was immersed in a 5 % lemon juice solution for 3 h, followed by 3 h of immersion in distilled water. The other group was immersed in distilled water for 6 h as a control. The excess moisture was then removed using a spin-drier (Xiaoya Domestic Appliances Co. Ltd., Jinan, Shandong, China), and the samples were vacuum-packed in polyethylene bags. The packaged samples were then cooked at 100 °C for 20 min and cooled at room temperature for further analysis.

### 2.4 pH measurement

Potato slices of different sizes (small, medium, and large) 5 g each were homogenized in a 50 mL deionized water and filtered through Whatman No. 4 filter paper, and the filtrate was utilized for pH analysis using a UB-5 UltraBasic pH meter (Denver Instrument Company) (Tsouvaltzis & Brecht, 2017).

### 2.5 Sensory evaluation

Fourteen sensory panelists in the field of food science (7 men and 7 women, ages 30 to 45) were selected voluntarily, with the understanding that the samples under evaluation were safe. All participants provided signed informed consent, and their participation was entirely voluntary, with the understanding that this was an experimental study. The participants were regular consumers of similar products, and those with allergies to any ingredients in the test samples were excluded from the study. The potato slices were freshly prepared just before the assessment, uniformly cut, and consistently presented to the panelists to ensure reliable results. The codes assigned to each dish were randomized and handed to the participants simultaneously. After that, a 9-point Hedonic scale was employed to measure the degree of participants' liking or disliking of quality characteristics including appearance, mouthfeel, and overall acceptability. Each attribute was scored from 1 to 9 (1: extremely dislike; 9: extremely like). The average score for each attribute was calculated and recorded at the end of the evaluation process (Almoselhy, 2023).

### 2.6 Color measurement

Small-size, medium-size, and large-size potato tubers were sliced into 0.5 cm-thick slices using a potato slicer. Color parameters were evaluated using a tristimulus colorimeter (model DP-400 with chroma meter model CR-400, Konica Minolta Sensing, Inc., Osaka, Japan). The color values were expressed as L* (100 = white, 0 = black), a* (+a* = redness, ˗a* = greenness), and b* (+b* = yellowness, ˗b* = blueness). A standard white plate with reflectance values of L* = 93.68 was used as a reference. Three color measurements were taken from the center of each slice. The mean values, along with the standard deviations, were then reported. In addition, the total color variation index (*ΔE*), hue angle (*h*), quantitative attribute of colorfulness (chroma or saturation, C*), and browning index (*BI*) between cooked and raw samples were calculated using eqs. (1˗4) outlined by Abd El-Baset and Almoselhy (2023), with some modifications.

(1) $$\mathrm{\Delta E}=\sqrt{{\mathrm{\Delta L}}^{*2}+{\mathrm{\Delta a}}^{*2}+{\mathrm{\Delta b}}^{*2}}$$

(2) $$C^{*}=\sqrt{a^{*2}+b^{*2}}$$

(3) $$h=\tan^{-1}\left(\frac{a^{*}}{b^{*}}\right)$$

(4) $$\mathrm{BI}=\frac{100}{0.17}\;\left(\frac{a^{*}+1.75L^{*}}{5.645L^{*}+a^{*}-0.012b^{*}}-0.31\right)\;$$

Additionally, the potato slices were arranged on a white ceramic plate to minimize any color interference during image capture. They were then photographed using a Sony a7 III Mirrorless Camera (Co., Tokyo, Japan) under constant light-emitting diode (LED) lighting conditions.

### 2.7 Hardness measurement

The hardness of small-size, medium-size, and large-size potato slices (SML-Ps) was determined using a puncture test. A texture analyzing machine (TA.XT Plus, England Instrumentation System Co., TA, England) was used for the hardness measurement according to Zhao et al. (2017) with slight modifications. A Texture Profile Analysis (TPA) with a P/6 probe was used. The instrument parameter settings were as follows: plotting parameter, 1.00 mm/s; testing speed, 1.00 mm/s; post-test speed, 1.00 mm/s; compression ratio, 30 %; and time between two compressions, 2 s. Each measurement was performed 3 times, and the average was obtained.

### 2.8 Microstructure studies

#### 2.8.1 Fluorescence microscopy (FM)

Several samples from the perimedullary parenchyma of the potatoes were cut to the appropriate size and fixed with 2 % (*w*/*v*) formaldehyde and 3 % (w/v) glutaraldehyde in 0.1 M phosphate buffer (pH 7.2) for 2 h at room temperature. After three washes in the same buffer, the samples were post-fixed with 1 % (w/v) OsO_4_ in the same buffer for 1 h at room temperature. The three buffer washes were repeated. They were then dehydrated using an acetone/water series (25 %, 50 %, 75 %, 95 %, and 100 %). The samples were then kept in each gradient for 10 min and in 100 % acetone for 2 h. Afterward, the samples were first embedded with a mixture of acetone and resin (Procure 812) in a 50 %:50 % ratio on a stirrer overnight, and then the acetone was replaced with fresh 100 % resin for another 8 h on the stirrer. Finally, the samples were molded in 100 % fresh resin at 60 °C for 48 h. Sections 1 μm in thickness were cut from trimmed resin blocks using a glass knife and ultramicrotome (Leica, Austria). They were then mounted onto a glass slide, stained with 0.05 % Toluidine Blue, and viewed under a fluorescence microscope (Olympus BX51, Japan) (Bordoloi et al., 2012).

#### 2.8.2 Scanning electron microscopy (SEM)

Morphological characterization was conducted using SEM (HITACHI SU-8010, Japan Instrumentation System Co., HITACHI, Japan). The samples were immersed in isopropyl acetate before critical-point drying using (HCP-2, Hitachi Koki Co., Ltd., Tokyo, Japan). Subsequently, the dried samples were placed in a carrier with carbon tape mounted on an aluminum sample holder and stabled by gold spraying (KYKY SBC-12 small ion sputter, Beijing Zhongquan Co., Ltd., Beijing, China) for 25 s at 5–7 Pa and were then scanned at 20 kV with a magnification of ×1000 (Zhao et al., 2017).

### 2.9 Determination of vitamin C content

Ascorbic acid content was measured using a method described by Gong et al. (2022) after some modifications. Briefly, a 10 g sample was mechanically stirred in a 50 mL metaphosphoric acid–acetic acid solution for 5 min and then centrifuged at 10,000 rpm for 15 min at 4 °C. Supernatant filtering was done using a 0.45 μm cellulose acetate filter before measuring. The ascorbic acid content was quantified by comparison with commercially available standards and expressed as (mg/g FW).

### 2.10 Total phenolics analysis

The total phenolics of each extract from SML-Ps were measured according to the method described by Homaida et al. (2017), with slight modifications. In brief, a phosphor-wolf-ramate phosphor-molybdate complex is reduced based on blue reaction products. Then, 300 μL of either standard sample was carefully transferred into a centrifuge tube. Subsequently, 1.5 mL of Folin-Ciocalteu reagent was added, followed by (1.2 mL) of Na_2_CO_3_ (7.5 %, *w*/*v*). Afterward, the solution was gently vortexed for a few seconds and immediately incubated for 30 min in a dark room. The mixture was then centrifuged at 10,000 rpm for 5 min. After that, the resulting absorbance was measured at a wavelength of 765 nm using a spectrophotometer (Spectro UV–vis Auto UV-2602, USA). A blank solution containing 80 % methanol was used as a reference. From the standard curve of gallic acid, the total phenol content was estimated and expressed in GAE, mg/100 g of dry weight.

### 2.11 Extraction by headspace solid-phase microextraction

In accordance with the method outlined by Shen et al. (2024) after some modifications, headspace solid-phase microextraction (HS-SPME) was employed to extract volatile compounds. 3 g from each sample was placed in a 10 mL headspace vial, together with 100 ppm of o-dichlorobenzene (dissolved in ethanol) as an internal standard. The vial was then promptly sealed and incubated at 60 °C for 15 min in a temperature-controlled oscillator operating at 500 rpm. Then, SPME-Arrow fibers coated with divinyl benzene/carbon wide range/polydimethylsiloxane (DVB/C-WR/PDMS; 110 μm × 20 mm), procured from CTC Analytics AG (Zwingen, Switzerland), were exposed to the headspace of the sample for 60 min, with a sample injection and desorption time of 280 s. The volatile compounds were then analyzed using GC–MS (Guangzhou Hexin Instrument Co., Ltd., GGT 0620, Guangzhou, China). The GC system comprised a primary chromatographic column (DB-5 ms: 30 m × 250 μm × 0.25 μm; Agilent Technologies) and a secondary chromatographic column (DB-17: 1.0 m × 180 μm × 0.18 μm; Agilent Technologies). The key gas chromatography parameters included a transfer line temperature of 280 °C, helium as the carrier gas (99.99 %) with a constant flow rate of 1 mL/min, and a non-split flow. The initial temperature of the chromatographic column was set at 40 °C for 3 min, followed by a ramp-up of 3 °C/min to 270 °C, and a hold time of 4 min. The primary mass spectrometry parameters consisted of an electron ionization source, a source temperature of 250 °C, a mass collection range of 40–500 amu, a collection rate of 101 spectra/s, and a solvent delay of 180 s. To facilitate the heating and cooling phases, a solid-state modulator SSM1820 (J&X Technologies, Shanghai, China) was placed between the two chromatographic columns. The modulator's main parameters involved a semi-volatile (SV) series modulation column (1.3 m × 0.25 mm; C6-C40) with a modulation period set to 4 s. Retention indices were calculated based on the retention times of normal alkanes (C7˗C30) under identical chromatographic conditions. The identification of volatile compounds was then compared with mass spectral data from the NIST library.

### 2.12 Infra-red spectroscopic analysis

The absorption spectra of the samples were obtained using Fourier transform infra-red (FTIR) spectroscopy (Thermo Electron Instruments Co., Ltd., Shanghai, China). The FTIR spectra of the dry alcohol insoluble residue (AIR) of potato samples were recorded between 400 and 4000 cm^˗1^ in a NICOET spectrometer. The absorption spectra of the samples were recorded using a KBr pallet containing 0.1 % sample (Jeddou et al., 2016).

### 2.13 Data processing and statistical analysis

All measurements were performed in three replicates, and the sensory evaluations were carried out in 14 replicates. Data were presented as mean ± standard deviation (SD). Tukey's post hoc test and analysis of variance (ANOVA) were conducted using Statistix Software (version 8.1, USA). The figures in this study were drawn using OriginPro version 2024 (Origin Lab, MA, USA).

## 3 Results and discussion

### 3.1 pH analysis

**Fig. 1:**
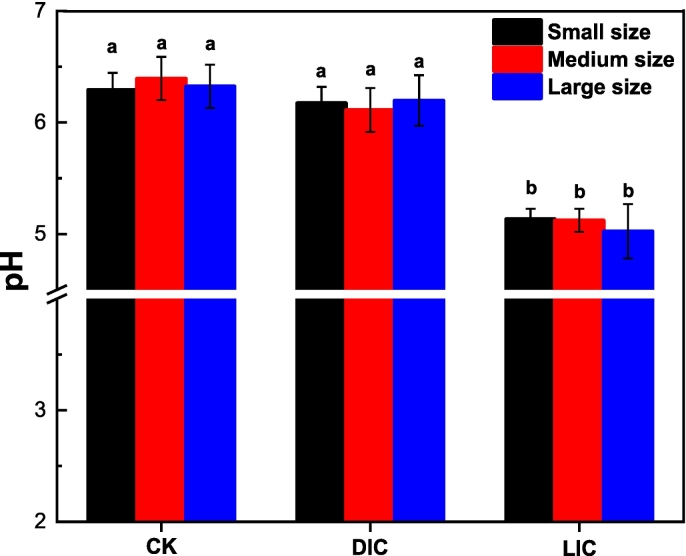
Changes in pH value of the treatment and control of SML-Ps before and after cooking. Significant differences at *P < 0.05* are indicated by different lowercase letters. The error bar represents the standard deviation (*n* = 3) between mean values. Fresh samples (CK); distilled water immersed cooked (DIC); lemon juice immersed cooked (LIC).

Lemon juice, rich in citric acid, is commonly used in food preparation and preservation (Vujčić Bok, Šola, & Rusak, 2022). As shown in Fig. 1, there were no significant differences in pH levels between fresh samples (CK) and those immersed in distilled water, distilled water immersed cooked (DIC). In contrast, samples immersed in a 5 % lemon juice solution, lemon juice immersed cooked (LIC), had significantly lower pH levels after cooking compared to CK and DIC. These findings are consistent with a study by Kita, Bråthen, Knutsen, & Wicklund, 2004, which revealed that soaking or blanching potato slices in acidic solutions like citric acid reduced the pH of potato juice due to the high acidity (low pH) of the treatment solutions used (Vujčić Bok, Šola, & Rusak, 2022). The slight pH changes resulting from the LJP are unlikely to impact consumer preferences. To confirm acceptability to consumers, a sensory evaluation was conducted.

### 3.2 Sensory analysis of CPS-ds

Sensory evaluation of cooked or baked products is important for assessing the acceptability of the final product by consumers (Abker et al., 2024; Almoselhy, 2023). The sensory attributes of appearance, mouthfeel, and overall acceptability were assessed within a range of 1 to 9. Table 1 **and Fig. S1** (refer to supplementary data) show slight differences (*P < 0.05*) in all sensory attributes. LJP samples were ranked best in terms of mouthfeel and overall acceptability. Conversely, there was no significant difference in appearance between the treatment and control samples. Based on the sensory evaluations, small-size lemon juice immersed cooked (SLIC) was the most acceptable. These findings collectively suggest that LJP plays a crucial role in enhancing the mouthfeel and overall acceptability of CPS-Ds.

**Table 1:** Sensory analysis and color values of small-size, medium-size, and large-size potato slices cooked at 100 °C for 20 min.

Treatment	Sensory evaluation			Color						
	Appearance	Mouthfeel	Acceptability	L*	a*	b*	*ΔE*	C*	H*	*BI*
SDIC	6.38 ± 0.93^a^	5.84 ± 0.17^b^	6.53 ± 0.28^b^	76.26 ± 0.44^b^	˗3.62 ± 0.46^a^	18.06 ± 0.78^a^	6.03 ± 2.39^a^	11.42 ± 3.04^b^	1.77 ± 0.06^a^	˗3.35 ± 0.31^b^
SLIC	6.97 ± 0.55^a^	6.46 ± 0.18^a^	7.89 ± 0.28^a^	73.42 ± 3.10^bc^	˗3.40 ± 0.70^a^	11.02 ± 0.74^c^	2.51 ± 2.39^c^	11.53 ± 3.04^a^	1.87 ± 0.06^a^	˗3.29 ± 0.31^b^
MDIC	6.93 ± 0.53^a^	5.85 ± 0.23^b^	6.96 ± 0.27^b^	75.58 ± 0.66^b^	˗3.97 ± 0.50^a^	11.20 ± 0.43^c^	5.98 ± 2.39b	11.85 ± 3.04^a^	1.91 ± 0.06^a^	˗3.75 ± 0.31^b^
MLIC	6.38 ± 0.52^a^	6.84 ± 0.23^a^	6.53 ± 0.33^b^	71.51 ± 0.84^c^	˗3.35 ± 0.72^a^	09.11 ± 0.13^d^	1.99 ± 2.39^d^	10.71 ± 3.04^d^	1.92 ± 0.06^a^	˗3.34 ± 0.31^b^
LDIC	6.96 ± 0.44^a^	5.83 ± 0.54^b^	6.99 ± 0.31^b^	82.61 ± 0.56^a^	˗3.79 ± 0.36^a^	13.16 ± 0.73^b^	5.78 ± 2.39^b^	11.70 ± 3.04^a^	1.85 ± 0.06^a^	˗3.26 ± 0.31^b^
LLIC	6.72 ± 0.43^a^	6.84 ± 0.37^a^	6.53 ± 0.44^b^	74.20 ± 0.58^bc^	˗2.91 ± 0.51^a^	11.08 ± 0.55^c^	1.92 ± 2.39^d^	11.46 ± 3.04^b^	1.83 ± 0.06^a^	˗2.78 ± 0.31^a^

Values are presented as mean ± standard deviation (n = 3); Values in the same column followed by different superscripts are significantly different at P < 0.05.

***Note*:** (SDIC) small-size distilled water immersed cooked; (SLIC) small-size lemon juice immersed cooked; (MDIC) medium-size distilled water immersed cooked; (MLIC) medium-size lemon juice immersed cooked; (LDIC) large-size distilled water immersed cooked; (LLIC) large-size lemon juice immersed cooked.

### 3.3 Color analysis of CPS-ds

Color is one of the main quality attributes that often affects consumers' perception of CPS, sometimes even more than flavor and texture. Consumers are visually drawn to it, and it greatly affects the acceptability of the final products (Abd El-Baset & Almoselhy, 2023; Izli et al., 2017). The color analysis of CPS-Ds is summarized in Table 1 **and Fig. S2** (refer to supplementary data). Among the various color indicators, the L* value ranged from 71.51 to 82.61. A higher L* value indicated a brighter potato slice. The control group with large-size potato slices, large-size distilled water immersed cooked (LDIC), exhibited the highest lightness (L* = 82.61) compared to the other groups. The a* value ranged from ˗3.97 to ˗2.91, and there were no significant differences in the a* value between the control and treatment samples. The a* value indicated that LJP did not significantly affect the greenness (a*) of potato slices of different sizes. The b* value indicated the level of yellowness or blueness in the samples. The b* range was 9.11 and 18.06, with higher b* values indicating a more pronounced yellow color in the sample. These results were consistent with previous research (Xiao, Bai, & He, 2020) and fell within the normal range. Additionally, the total color difference (*ΔE*) increased from 1.92, 1.99, and 2.51 for the large-size lemon juice immersed cooked (LLIC), medium-size lemon juice immersed cooked (MLIC), and SLIC, to 5.78, 5.98, and 6.03 for the large-size distilled water immersed cooked (LDIC), medium-size distilled water immersed cooked (MDIC), and small-size distilled water immersed cooked (SDIC), respectively. This indicated a slight decrease in the total color difference (*ΔE*) in the treatment samples. For chroma (C*), the values ranged from 10.71 to 11.42, suggesting slight changes in the color saturation of CPS-Ds. Hue angles (*h*) ranged from 1.77 to ˗1.99, indicating that all samples were in the same region. Browning Index (*BI*) values ranged from ˗3.75 to ˗2.78, suggesting slight changes in the browning index between the treatment and control samples. These findings suggest that LJP helps maintain the desirable color attributes of CPS-Ds, which are crucial for consumer appeal.

### 3.4 Hardness analysis

**Fig. 2:**
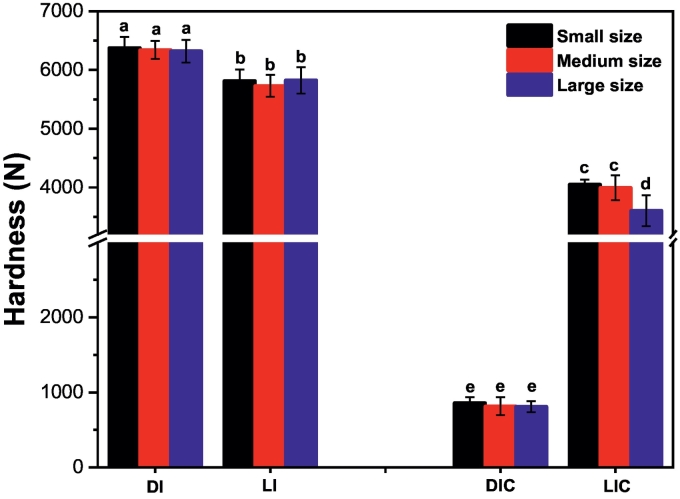
Changes in the hardness of the treatment and control of SML-Ps before and after cooking. Significant differences at *P < 0.05* are indicated by different lowercase letters. The error bar represents the standard deviation (n = 3) between mean values.

Hardness is an important quality factor for plant-based food. It affects the shelf life of fruits and vegetables, as well as consumer acceptance and demand (Liu et al., 2022; Moens et al., 2021). Fig. 2 illustrates the evaluation of hardness before and after cooking. Before cooking, there were slight differences in hardness between the control samples, distilled water immersed (DI), and the treatment samples, lemon juice immersed (LI), indicating that LJP did not significantly alter the raw texture of the SML-Ps. However, after cooking, the control group (SDIC, MDIC, and LDIC) showed a noticeable reduction in hardness, with values of 857.73 N, 815.63 N, and 809.93 N, respectively. In contrast, the treatment samples retained a higher level of firmness after cooking. SLIC, MLIC, and LLIC had hardness values of 4051.09 N, 3994.76 N, and 3603.66 N, respectively. Similar results were observed in potatoes and lotus rhizomes, where acetic acid pretreatment prevented tissue softness, resulting in an intermediate hardness even after thermal processing (Liu et al., 2020; Zhao et al., 2017). Overall, high hardness is not favorable for the product due to consumer acceptance. However, low hardness is considered less safe due to higher moisture content. Therefore, an intermediate hardness level is preferred as it maintains an acceptable level of hardness while having a lower moisture content (Abd El-Baset & Almoselhy, 2023). These results suggest that LJP helps retain the firmness of CPS-Ds, balancing consumer preferences and safety concerns.

### 3.5 Microstructure studies

#### 3.5.1 Fluorescence microscopy

**Fig. 3:**
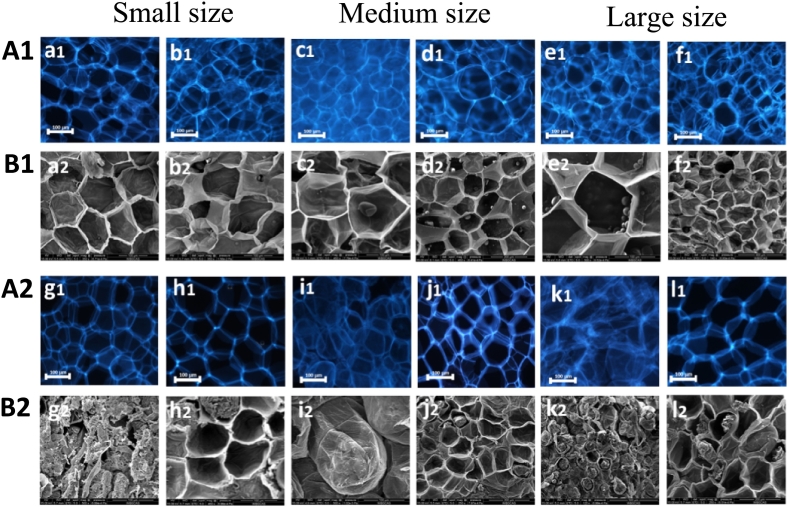
Fluorescence microscope (**A1, A2**) and SEM (**B1, B2**) images of SML-Ps before and after cooking. Small-size: distilled water (a1, a2), lemon juice (b1, b2), cooked distilled water (g1, g2), cooked lemon juice (h1,h2). Medium-size: distilled water (c1, c2), lemon juice (d1, d2), cooked distilled water (i1, i2), cooked lemon juice (j1, j2). Large-size: distilled water (e1, e2), lemon juice (f1, f2), cooked distilled water (k1,k2), cooked lemon juice (l1,l2).

Fluorescence microscopy (FM) is a valuable tool for research on the cell wall and cellular structure and function (Sengupta et al., 2012). FM micrographs revealed differences in the microstructure of slice parenchyma among the treatment and control samples. The raw potato slice parenchyma from all the samples showed preservation of the polygonal cell wall outline, but some cells lost their contents during sample preparation (Bordoloi et al., 2012). Figs. 3 **(A1 and A2)** display the FM micrographs before and after cooking. As shown in Fig. 3 **A1**, before cooking, the results indicated that potato slices in the control and treatment groups had intact cell walls (a1, b1, c1, d1, e1, and f1). However, after cooking, the results showed that potato slices in the treatment group had more intact cell walls as shown in Fig. 3 **A2** (h1, j1, and l1) than those in the control group (g1, i1, and k1). These findings suggested that pretreatment with a 5 % lemon juice solution may have a protective or stabilizing effect on the cell wall structure of CPS-Ds. Therefore, this is well matched with the findings of Zhao et al. (2017), who found that acetic acid pretreatment can potentially preserve the cell wall integrity and the texture of CPS by modifying the cell wall polysaccharides.

#### 3.5.2 Scanning electron microscopy (SEM)

As shown in Fig. 3 **(B1 and B2)**, SEM was used to examine the cell wall structure of potato slices in the control and treatment groups. Before cooking, potato slices in both the control and treatment groups showed intact cell walls (a2, b2, c2, d2, e2, and f2). However, the cooking process affected the tricellular junction region and the entire cell wall in the control group, causing a filamentous and flocculent appearance, as seen in Fig. 3 **B2** (g2, i2, and k2). These results are consistent with the decreased hardness observed and confirmed by the SEM and FM analysis, which showed the thickness and shrinkage of the control potato slices' cell walls. A study conducted by Gonzalez & Barrett, 2010 stated that the main factors contributing to the reduction in tissue rigidity are the loss of cell turgor and adhesion with adjacent cells. Additionally, Liu, et al. (2020) reported that the integrity of cell walls is compromised due to the thermal breakdown of polysaccharides. In contrast, the treatment group, as shown in **Fig. B2** (h2, j2, and l2), displayed a relatively preserved and intact cell wall structure, which is the key factor in maintaining the hardness of the cooked tissue. These findings indicated that the integrity of the cell wall structure of CPS-Ds, particularly when samples are pretreated with a 5 % lemon juice solution, is closely related to the texture of its cell wall. Overall, the FM and SEM images demonstrated that LJP strengthens the cell wall integrity of the CPS-Ds, resulting in improved hardness.

### 3.6 Ascorbic acid analysis

**Fig. 4:**
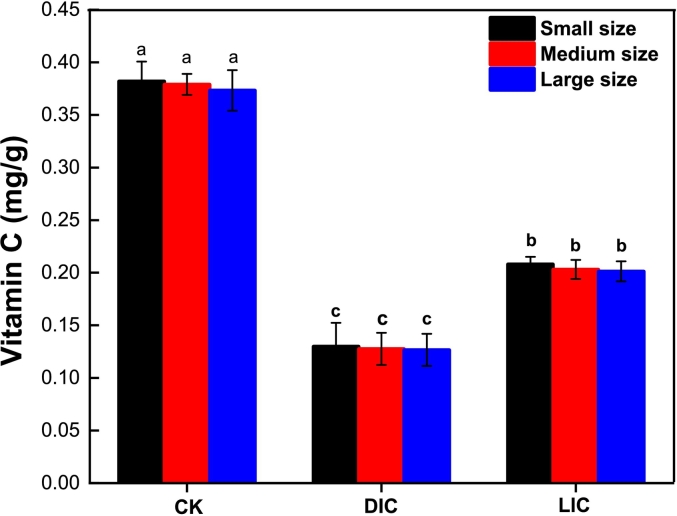
Ascorbic acid content in the treatment and control of SML-Ps before and after cooking. Significant differences at *P < 0.05* are indicated by different lowercase letters. The error bar represents the standard deviation (n = 3) between mean values. Vitamin C (ViC); Fresh (CK); distilled water immersed cooked (DIC); lemon juice immersed cooked (LIC).

Fruits and vegetables are often measured by their ascorbic acid content, which is an important indicator of nutritional quality (Gong et al., 2022). Fig. 4 shows the variations in ascorbic acid content between the treatment and control samples. Initially, the CK had the highest ascorbic acid content before cooking, at around 0.378 mg/g. Nevertheless, after cooking, the LIC experienced a significant increase in ascorbic acid levels (0.2042 mg/g) compared to the DIC (0.128 mg/g). Lemon juice, with its antioxidant capacity and low pH, may help preserve the existing ascorbic acid content in potatoes during cooking by inhibiting degradation reactions (Vujčić Bok, Šola, & Rusak, 2022). These findings collectively indicated that LJP not only helps preserve ascorbic acid content during thermal treatments but also enhances the ascorbic acid content in CPS-Ds, thereby improving their nutritional profile.

### 3.7 Total phenolic analysis

**Fig. 5:**
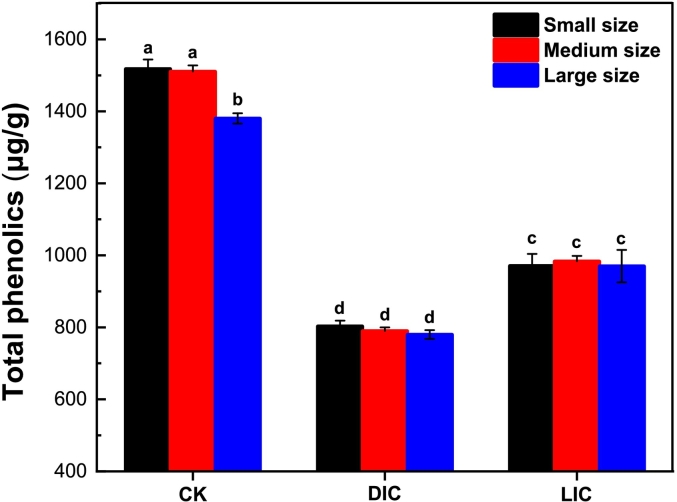
Total phenols in the treatment and control of SML-Ps before and after cooking. Significant differences at *P < 0.05* are indicated by different lowercase letters. The error bar represents the standard deviation (n = 3) between mean values.

Phenolic compounds have various biological and functional activities that contribute to the quality of fruits and vegetables (Homaida et al., 2017). As shown in Fig. 5, CK had the highest concentration of total phenolic compounds, approximately 1469.67 μg/g. However, after cooking, there was a significant decrease in total phenolics, dropping to around 791.11 μg/g in the control group (DIC). This decrease may be due to the sensitivity of phenolic substances to heat treatments (Fang et al., 2022; Tian et al., 2016). While potato slices when immersed in a 5 % lemon juice solution and cooked (LIC), the total phenolics were preserved at a high level of about 974.792 μg/g compared to the DIC. This preservation may be attributed to the highly acidic nature of lemon juice, which typically has a pH of around 2˗3. Therefore, the acidity of lemon juice stabilizes phenolic compounds and prevents their degradation during the cooking process. Lemon juice also contains antioxidants that inhibit oxidation reactions, which can breakdown phenolic compounds during heating. Additionally, the low pH of lemon juice disrupts hydrogen bonding and hydrophobic interactions between polyphenols and lipids (Vujčić Bok, Šola, & Rusak, 2022). Understanding the dynamics of phenolic content in CPS-Ds provides valuable insights into the impact of LJP on their bioactive compounds. Further research could explore the specific mechanisms involved in the preservation of phenolic compounds during LJP and the cooking process.

### 3.8 Volatile compounds of CPS-ds analyzed by GC–MS

The aroma of cooked potatoes differs significantly from that of raw potatoes due to various volatile compounds produced during cooking. These compounds are generated through thermal pyrolysis processes, including terpene glycosidic bond release, carotenoid degradation, caramelization, Maillard reactions, and Strecker degradation (Jiang et al., 2023; Tsai et al., 2021). This study identified 20 volatile compounds in CPS-Ds, immersed in either distilled water or lemon juice, using GC–MS, as summarized in Table 2 **and Fig. S3 (A-F)** in the supplementary data. The control samples (SDIC, MDIC, LDIC) generally exhibited a higher number of volatile compounds compared to the treatment samples (SLIC, MLIC, LLIC). For instance, compounds such as furan, 2-pentyl, undecanol, decanal, 3-phenol-propanol, cyclohexane, dichloromethane, and methyl allyl disulfide were predominantly found in the control samples. This suggests that the absence of acidic or antioxidant components allows for more complex thermal degradation pathways, leading to a broader range of volatile compounds. Conversely, compounds such as pentanol, pentadecane, and hexadecane were more prominent in the treatment samples. This indicates that lemon juice can significantly alter the volatile profile of cooked potatoes by enhancing certain desirable compounds and reducing others that may contribute to off-flavors (Aisala, Sola, Hopia, Linderborg, & Sandell, 2019). The presence of these compounds in the lemon juice treatments aligns with findings by Jayanty, Diganta, & Raven, 2019, who noted that natural additives like lemon juice can modify flavor profiles, potentially enhancing the sensory qualities of cooked products. This is consistent with the findings of Tsai et al. (2021), who suggested that natural additives like lemon juice may not entirely prevent off-flavors but can enhance the taste profile of cooked products.

**Table 2:** Volatile compounds of cooked potato slices of different sizes identified by GC–MS.

Peak No	Compound name	Formula	RT	SDIC	SLIC	MDIC	MLIC	LDIC	LLIC
1	1-Pentanol	C5H12O	4.28	ND	3.16	ND	2.17	ND	ND
2	Hexanol	C6H14O	6.76	5.44	3.13	4.42	2.13	3.38	ND
3	Furan, 2-pentyl	C9H14O	11.79	7.26	ND	6.28	ND	5.27	ND
4	Octanal	C8H16O	12.18	10.12	3.28	9.11	2.31	8.91	1.26
5	1-Hexanol, 2-ethyl	C8H18O	12.92	7.72	9.57	6.71	8.58	5.72	7.58
6	Benzene	C6H6	13.94	1.07	0.94	0.83	0.25	0.14	0.32
7	Carveol	C10H16O	14.47	1.58	0.53	0.57	ND	0.46	0.84
8	Undecanol	C11H24O	15.56	3.96	ND	2.96	ND	1.98	ND
9	2,4-Dimethyl	C7H16	16.02	ND	ND	9.46	ND	6.53	ND
10	β-Pinene	C10H16	17.02	ND	ND	0.32	ND	ND	ND
11	Decanal	C10H20O	18.44	6.58	ND	0.73	ND	ND	ND
12	3-Phenolpropanol	C9H12O	19.97	0.95	ND	ND	ND	0.86	ND
13	Pentadecane	C15H32	20.27	ND	2.15	ND	1.16	ND	0.13
14	Hexadecane	C16H34	20.34	ND	1.73	ND	0.75	ND	0.65
15	Dodecane	C12H26	21.13	4.52	2.13	3.48	1.17	2.46	ND
16	Cyclohexane	C6H12	21.23	1.31	ND	0.78	ND	0.58	ND
17	Butylated	C15H24O	23.26	5.57	14.86	4.56	13.89	3.53	12.83
18	6-Methyl-5-hepten	C8H14O	25.91	2.79	1.94	1.14	0.58	0.84	0.46
19	Dichloromethane	C2Cl2	26.28	4.08	ND	ND	ND	ND	ND
20	Methyl allyl disulfide	C4H8S2	29.24	4.94	ND	ND	ND	ND	ND

***Note*:** RT = Retention time; ND = not detected. (SDIC) small-size distilled water immersed cooked; (SLIC) small-size lemon juice immersed cooked; (MDIC) medium-size distilled water immersed cooked; (MLIC) medium-size lemon juice immersed cooked; (LDIC) large-size distilled water immersed cooked; (LLIC) large-size lemon juice immersed cooked.

### 3.9 Infra-red spectroscopic analysis

**Fig. 6A and B:**
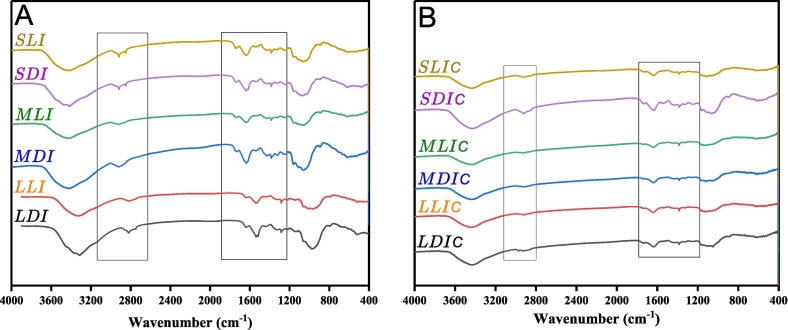
FT-IR spectrometry of AIR from potato slices before and after cooking. (SDI) small-size distilled water immersed; (SDIC); small-size distilled water immersed cooked; (SLI) small-size lemon juice immersed; (SLIC) small-size lemon juice immersed cooked; (MSDI) medium-size distilled water immersed; (MDIC) medium-size distilled water immersed cooked; (MLI) medium-size lemon juice immersed; (MLIC) medium-size lemon juice immersed cooked; (LDI) large-size distilled water immersed; (LDIC) large-size distilled water immersed cooked; (LLI) large-size lemon juice immersed; (LLIC) large-size lemon juice immersed cooked.

The Fourier transform infra-red spectroscopy was performed in the region of 4000 to 400 cm^˗1^ for the dried AIR of SML-Ps. As shown in Fig. 6A, before cooking, the SDI exhibits a prominent peak at ∼2922 cm^˗1^, indicating an aliphatic C—H stretch (Jagadeesan, Govindaraju, & Mazumder, 2020). The SLI also shows this peak with lower transmittance, suggesting increased absorption or interaction with lemon juice components. MDI and MLI display similar peaks at ∼2922 cm^˗1^ and 2853 cm^˗1^, respectively. LDI and LLI exhibit an aliphatic C˗H stretch, with LLI showing more intense absorption at ∼3413 cm^˗1^, indicating an O˗H stretch, likely due to a 5 % lemon juice solution. After cooking, as shown in **Fig. 6B**, the control samples (SDIC, MDIC, LDIC) show a decrease in the O˗H stretch peak at ∼3413 cm^˗1^, likely due to the loss of hydroxyl groups or reduced hydrogen bonding (Jeddou et al., 2016). Treatment samples (SLIC, MLIC, LLIC) exhibit a similar trend, but the O˗H peak remains more intense, suggesting that LJP may protect these functional groups or influence the cooking reaction.

## 4 Conclusion

The study assessed the impact of LJP on the texture and nutritional quality of CPS-Ds. The results indicated that pre-treating potato slices with lemon juice solution and cooking improved their texture, vitamin C content, and total phenolics. Sensory evaluations rated the SLIC sample highest for acceptability. Microstructural analysis (FM and SEM) demonstrated that LJP preserved the cell wall integrity of CPS-Ds. FT-IR spectra indicated no new functional groups were generated. Colorimetric values and GC–MS results revealed minimal effects on color and volatile compounds, while the pH decreased due to the lemon juice solution's acidity. This research guides manufacturers seeking to produce high-quality and time-efficient potato slices using the LJP method. Overall, LJP is a simple, safe, and cost-effective approach for industrializing potato processing. Further studies should investigate the shelf life and functional characteristics of CPS-Ds pre-treated with lemon juice solution.

## Funding

This research received no financial support.

## Ethical approval

Informed consent was obtained from all participants in the sensory evaluations conducted as a part of this study. While ethical permission for sensory panel research is not customary for either our country or our institution, we affirm that appropriate measures were taken to ensure the welfare and rights of participants throughout the evaluation process.

## CRediT authorship contribution statement

**Alsadig Yahya:** Writing – review & editing, Writing – original draft, Visualization, Software, Resources, Project administration, Methodology, Investigation, Funding acquisition, Formal analysis, Data curation, Conceptualization. **Abdeen Elkhedir:** Writing – review & editing, Software, Resources, Data curation. **Mamoun A. Homaida:** Software, Formal analysis, Conceptualization. **Ikhlas Galal-Eldin:** Writing – review & editing, Visualization, Software, Methodology, Investigation, Data curation. **Yassin Taha:** Writing – review & editing, Visualization, Formal analysis, Data curation. **Ezzalden Saleh:** Writing – review & editing, Visualization, Software, Investigation.

## Declaration of competing interest

The authors declare that they have no known competing financial interests or personal relationships that could have appeared to influence the work reported in this paper.

## Data Availability

Data will be made available on request.

## Appendix A Supplementary data

Supplementary material Image 1
