# Supplementary material for: Lemon juice pretreatment as a strategy to preserve the quality and enhance the texture of cooked potato slices of different sizes
Source: Food Chem X. 2024 Sep 2;24:101800. doi: 10.1016/j.fochx.2024.101800 (PMC11415885; doi:10.1016/j.fochx.2024.101800)
Supplement: Supplementary file 1 — Supplementary material [file mmc1.docx]

**Supplementary materials**

**
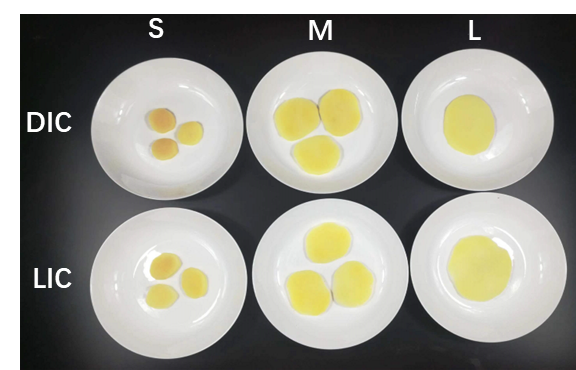
**

**Fig. S1.** Sensory evaluation of cooked potato slices for treatment and control groups. Distilled water immersed cooked (DIC); lemon juice immersed cooked (LIC); small-size (S); medium-size (M); large-size (L).


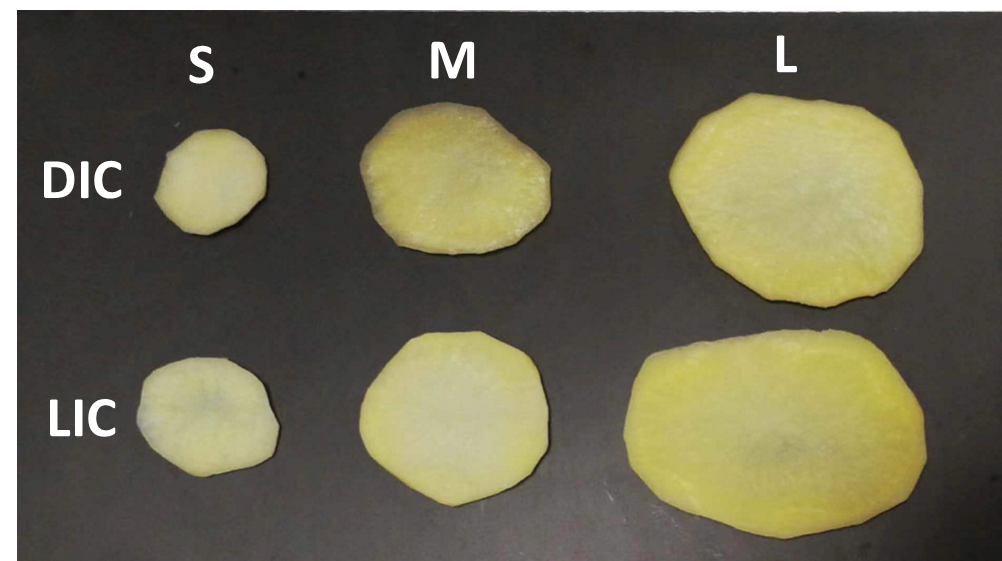


**Fig. S2.** Color changes in the treatment and control groups of SML-Ps after cooking. Distilled water immersed cooked (DIC); lemon juice immersed cooked (LIC); small-size (S); medium-size (M); large-size (L).

| 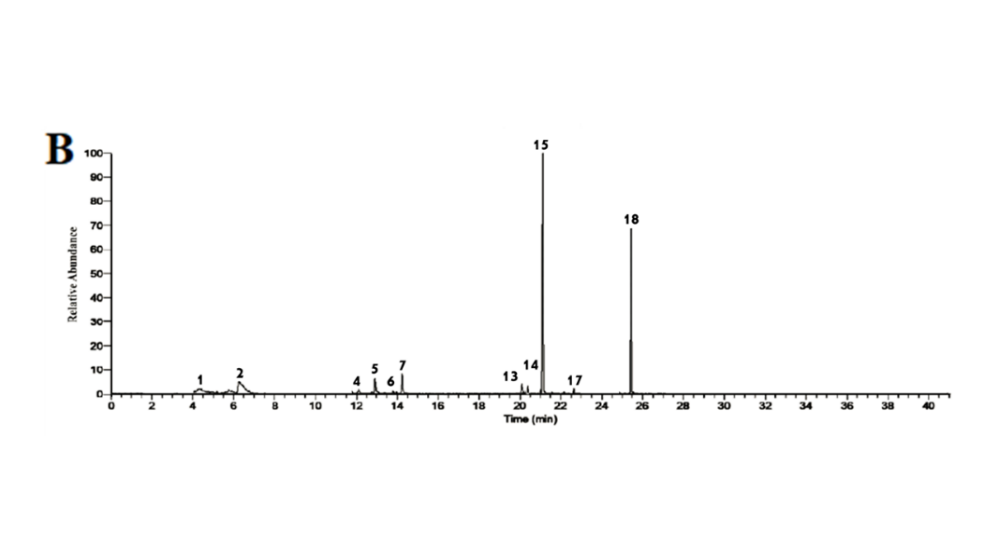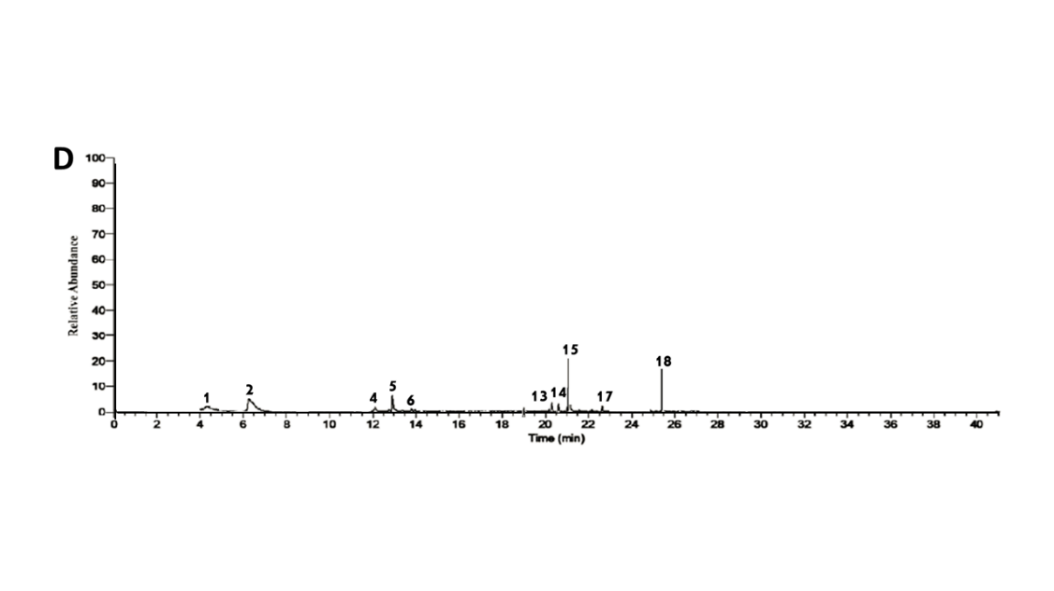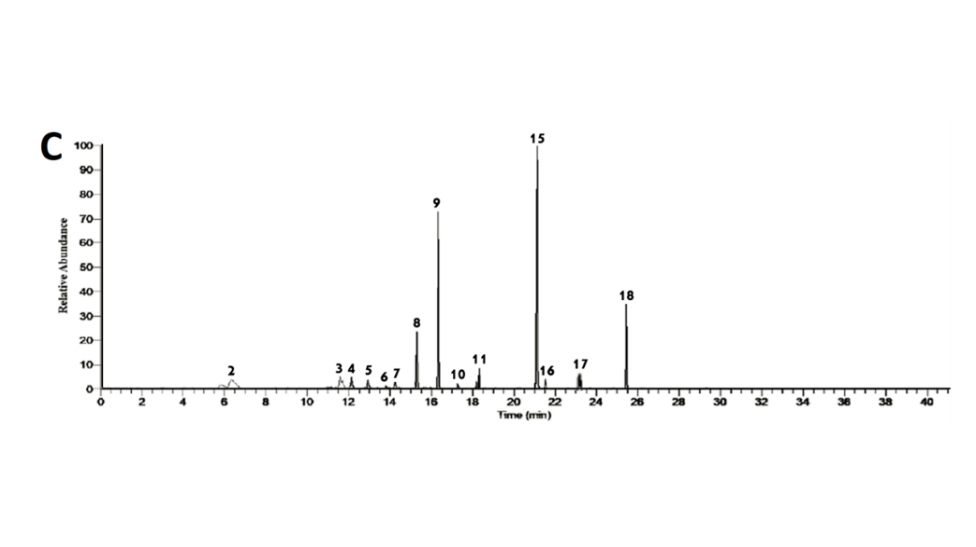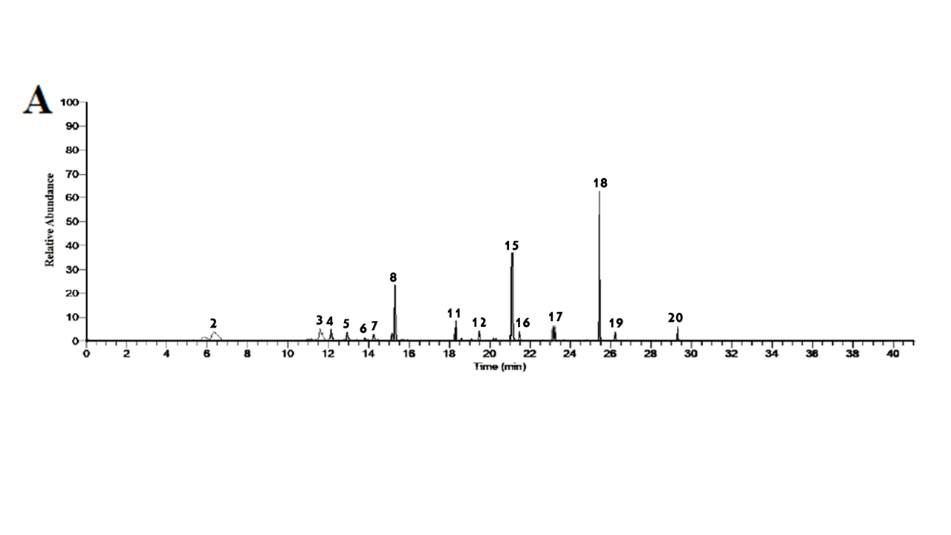  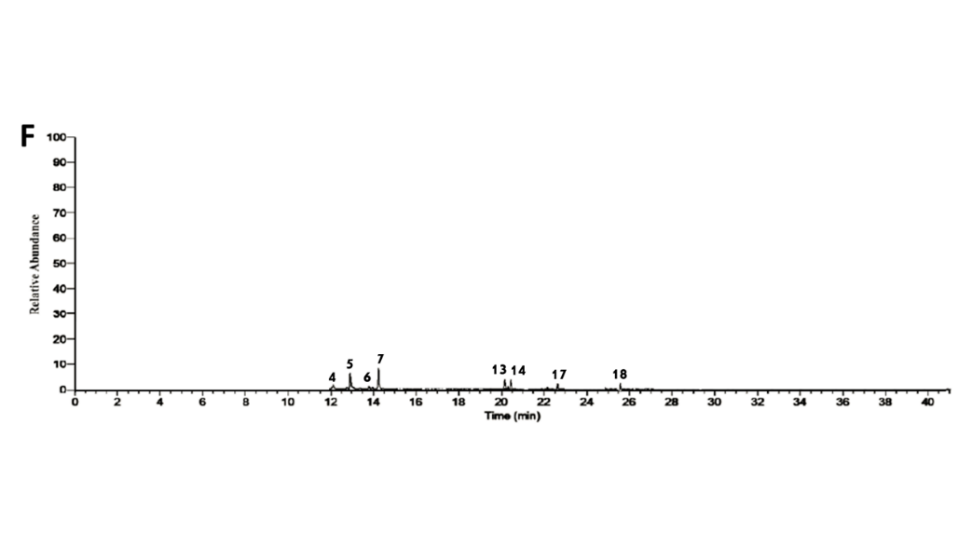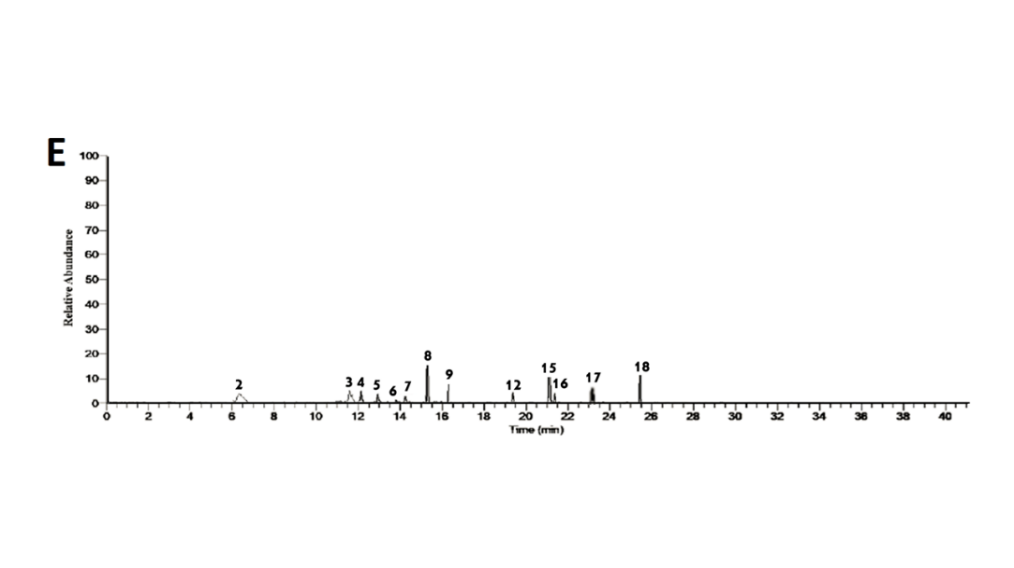 |
| --- |

**Fig. S3 (A-F)** Volatile compounds of cooked potato slices of different sizes identified by GC-MS. (**A**) small-size distilled water immersed cooked; (**B**) small-size lemon juice immersed cooked; (**C**) medium-size distilled water immersed cooked; (**D**) medium-size lemon juice immersed cooked; (**E**) large-size distilled water immersed cooked; (**F**) large-size lemon juice immersed cooked.
